# Interim Vaccine Effectiveness Against Influenza and Hospitalization, Republic of Korea, 2024–2025 (HIMM Network)

**DOI:** 10.3390/vaccines13111100

**Published:** 2025-10-28

**Authors:** Yu Jung Choi, Joon Young Song, Seong-Heon Wie, Jacob Lee, Jin-Soo Lee, Hye Won Jeong, Joong Sik Eom, Jang Wook Sohn, Won Suk Choi, Eliel Nham, Jin Gu Yoon, Ji Yun Noh, Hee Jin Cheong

**Affiliations:** 1 Division of Infectious Diseases, Department of Internal Medicine, Korea University College of Medicine, Seoul 02841, Republic of Korea; 2 Vaccine Innovation Center-KU Medicine (VIC-K), Seoul 02841, Republic of Korea; 3 Division of Infectious Diseases, Department of Internal Medicine, St. Vincent’s Hospital, College of Medicine, The Catholic University of Korea, Suwon 16247, Republic of Korea; 4 Division of Infectious Diseases, Department of Internal Medicine, Kangnam Sacred Heart Hospital, Hallym University College of Medicine, Seoul 07441, Republic of Korea; 5 Division of Infectious Diseases, Department of Internal Medicine, Inha University School of Medicine, Incheon 22332, Republic of Korea; 6 Department of Internal Medicine, Chungbuk National University College of Medicine, Cheongju 28644, Republic of Korea; 7 Division of Infectious Diseases, Department of Internal Medicine, Gil Medical Center, Gachon University College of Medicine, Incheon 21565, Republic of Korea

**Keywords:** influenza, influenza vaccine, vaccine effectiveness

## Abstract

**Background**: Influenza cases have surged earlier than usual during the 2024–2025 season, with A/H1N1 (pdm09) being the dominant strain. We aimed to investigate early estimates of influenza vaccine effectiveness (VE) for the 2024–2025 season to enhance our influenza response strategies. **Methods**: From November 1 to December 31, 2024, we enrolled 990 individuals with influenza-like illness from the hospital-based influenza surveillance network (Hospital-Based Influenza Morbidity and Mortality, HIMM), which consists of eight hospitals. **Results**: The overall adjusted VE was estimated to be −0.5% (95% confidence interval [CI], −34.0 to 24.6), with 0.4% (95% CI, −33.2 to 25.5) for influenza A. Analyses by influenza subtype were exploratory, given the limited number of subtyped cases. Although ineffective in preventing laboratory-confirmed influenza, influenza vaccination reduced influenza-related hospitalizations by 31.9% (95% CI, 3.5 to 51.9). **Conclusions**: It is necessary to enhance influenza vaccine effectiveness by selecting better-matched vaccine strains and introducing immune-enhanced vaccines.

## 1. Introduction

Despite the widespread focus on COVID-19, influenza still remains the respiratory virus with the greatest disease burden among the elderly [1,2]. During the COVID-19 pandemic, multiple social factors, such as the widespread use of face masks, social distancing, and hand hygiene, contributed to the suppression of various respiratory viruses. As a result, the prolonged reduction in viral exposures led to the accumulation of a susceptible population and weakening of herd immunity, which contributed to the earlier seasonal resurgence of respiratory viruses such as influenza and respiratory syncytial virus (RSV) after the pandemic [3]. During the last week of 2024 in South Korea, the Influenza-like illness (ILI) rate surged to 73.9 cases per 1000 persons, a 136% increase from the previous week’s 31.3, reaching the highest level in eight years. According to the Korea Disease Control and Prevention Agency (KDCA), as of week 52 (28 December 2024), the influenza positivity rate among patients with ILI was 50.9%. Among laboratory-confirmed influenza cases, the dominant strain was A/H1N1, which accounted for 68.0%, followed by A/H3N2 (29.3%) and influenza B (2.7%) [4]. Fortunately, the circulating strains are similar to the vaccine strains antigenically, indicating that the vaccine is likely to provide sufficient protection, with a vaccination rate of 80.0% (8.22 million people) in South Korea during this season [5]. In previous analyses of vaccine effectiveness (VE) over the past years, we observed significant variations by virus type as well as geographic region [6,7,8]. Given these factors, we aimed to investigate early estimates of influenza vaccine effectiveness for the 2024–2025 season to improve our influenza response strategies.

## 2. Materials and Methods

### 2.1. Study Design and Data Collection

We conducted a test-negative case–control study using the hospital-based influenza surveillance network (Hospital-Based Influenza Morbidity and Mortality, HIMM), which consists of eight hospitals geographically distributed across South Korea (three in Seoul, two in Gyeonggi, two in Incheon, and one in Chungcheongbuk-do). We enrolled patients who met the following criteria: (1) those who visited the emergency department or outpatient clinic for influenza-like illness (ILI), and (2) those who were hospitalized and underwent laboratory testing for influenza. ILI was defined as individuals with a sudden onset of fever ≥ 37.8 °C, accompanied by cough, sore throat, or rhinorrhea/nasal congestion [9]. We analyzed the early seasonal data based on diagnosis dates from 1 November to 31 December 2024. Influenza diagnosis was performed on nasopharyngeal swab specimens collected within seven days of symptom onset using commercially available rapid antigen test (RAT) and real-time reverse-transcription polymerase chain reaction (RT-qPCR) kits according to standard protocols [10]. Viral RNA extraction and amplification followed the recommended procedures, with appropriate controls included to ensure test validity.

### 2.2. Vaccination Information

In South Korea, the WHO Northern Hemisphere-recommended quadrivalent vaccine strains were administered, which included A/Victoria/2570/2019 (H1N1) pdm09-like virus, A/Thailand/8/2022 (H3N2)-like virus, B/Austria/1359417/2021 (B/Victoria lineage)-like virus, and B/Phuket/3073/2013 (B/Yamagata lineage)-like virus [11]. Vaccination information was collected through personal questionnaires, medical records, and the national immunization registry of the Korea Disease Control and Prevention Agency, with vaccinations considered valid if administered 14 days before diagnosis.

### 2.3. Statistical Analysis

Vaccine effectiveness (VE) was estimated using a test-negative design. Cases were defined as participants with laboratory-confirmed influenza, and controls were matched participants who tested negative for influenza. Each test-positive case was matched with one test-negative control based on age, sex, and date of specimen collection (1:1 ratio). Odds ratios (ORs) were calculated by comparing the odds of vaccination between case and control groups using conditional logistic regression, with adjustment for comorbidities. VE was calculated as 100 × (1 − OR). To minimize false-negative results, we restricted the controls to patients who visited the same hospital with ILI within 48 h of symptom onset. Because RATs have lower sensitivity compared with RT-qPCR, which could lead to misclassification bias, we additionally performed a sensitivity analysis including only RT-qPCR–tested participants. To assess the vaccine effectiveness in preventing severe outcomes, we evaluated the proportion of hospitalized patients whose admissions were attributable to influenza. Using conditional logistic regression adjusted for comorbidities, we conducted a test-negative design analysis among hospitalized patients. A *p*-value of <0.05 was considered statistically significant. All statistical analyses were performed using SPSS software, version 20.0.

### 2.4. Ethics Statement

This study was approved by the Institutional Review Board of each participating hospital, as listed in the Institutional Review Board Statement section. Written informed consent was obtained from all participants prior to enrollment.

## 3. Results

A total of 990 individuals were enrolled during the study period, including 495 test-positive cases and 495 test-negative controls (Supplementary Figure S1). Although rapidity and cost issues limited RT-qPCR testing to 239 subjects (24.1%), the results were cross-checked with RAT findings, and the result was considered positive if either test was positive. Among the influenza-positive cases, 96.6% (479/495) were identified as influenza A, 3.4% (17/495) were influenza B, and one case showed co-infection with both A and B. Among those who underwent RT-qPCR testing, influenza A/H1N1 was the predominant strain, comprising 71.7% (38/53) of cases subtyped by RT-PCR. These trends are consistent with those of national surveillance data, as illustrated in the pie chart in Supplementary Figure S2. There were two cases of co-infection: one where both A and B were detected in RAT, and the other where both A(H1N1) and A(H3N2) were detected simultaneously in RT-qPCR.

The baseline characteristics between the two groups are presented in Table 1. Most of the study population (98.0%) was enrolled in December, and 172 individuals (34.7%) in each group were aged 65 years or older. Overall, the case group had a higher proportion of individuals without underlying comorbidities (53.7% vs. 42.4%, *p* <0.001); however, there was no significant difference between the two groups for each comorbidity, except for solid malignancy (17.4% vs. 9.7%, *p* < 0.001). Vaccination rates were slightly higher in the case group (41.4%) compared to the control group (39.6%), but the difference was not statistically significant. Among the 401 vaccinated individuals, 237 (59.1%) received the standard quadrivalent egg-based influenza vaccines, while 164 (40.9%) received the cell-based vaccines. All clinical outcomes, except for mortality, were significantly worse in the control group.

The adjusted overall vaccine effectiveness (VE) was estimated to be −0.5% (95% confidence interval [CI], −34.3 to 24.4), with age-stratified estimates of −15.3% (95% CI, −66.7 to 20.3) for the 19–64-year age group and 18.5% (95% CI, −30.7 to 49.2) for the ≥65-year age group, none of which were statistically significant. Subtype-specific and age-stratified analyses are presented in Table 2, which are exploratory and underpowered due to the limited number of subtyped cases. Vaccine effectiveness estimates in the RT-qPCR–tested subgroup (n = 239) were not statistically significant (Supplementary Table S1). In particular, only two cases of influenza B were detected by RT-qPCR, precluding meaningful analysis for this subgroup.

Although vaccination did not show a significant effect on influenza incidence, it was associated with a protective effect against severe outcomes. In the overall population, individuals who received the influenza vaccine had a 31.9% reduced risk of hospital admission (95% CI, 3.5 to 51.9; *p* = 0.031). Intensive care unit admission and mortality rates were also lower among vaccinated individuals (Table 3).

## 4. Discussion

We estimated the real-world effectiveness of influenza vaccination during early periods of the 2024–2025 season. Unexpectedly, the VE in our study was estimated to be lower than anticipated. Unfortunately, we were unable to obtain genetic sequencing data to determine the match between the isolated viruses and the vaccine strain in the interim analysis. Since antigenic similarity does not fully explain VE, further analysis through genetic sequencing is needed. In addition, due to the reduction in natural influenza infections during the COVID-19 pandemic, it is possible that influenza vaccine effectiveness decreased in the post-pandemic period [12,13]. Nevertheless, vaccination remains the most reliable strategy for reducing the disease burden of influenza [14]. Although the VE against laboratory-confirmed influenza was lower than expected, influenza vaccination has been shown to prevent influenza-related hospitalizations and deaths. The Advisory Committee on Immunization Practices of the U.S. Centers for Disease Control and Prevention recommends that adults aged 65 years and older receive highly immunogenic vaccines, either high-dose or adjuvanted influenza vaccine, as a priority [15]. In South Korea, the MF59-adjuvanted influenza vaccine (Fluad^®^, CSL Seqirus, Parkville, Australia) was approved in 2022, and the high-dose influenza vaccine (Efluelda^®^, Sanofi, Paris, France) for adults aged 65 years and older received approval in 2023 [16]. In a randomized controlled trial, the high-dose influenza vaccine demonstrated a 24.2% (95% CI, 9.7–36.5) higher relative effectiveness against laboratory-confirmed influenza compared to the standard-dose influenza vaccine [17]. Similarly, a meta-analysis showed that the MF59-adjuvanted influenza vaccine provided a 14% higher relative effectiveness in preventing influenza compared to the unadjuvanted standard-dose influenza vaccine [18]. However, in this study, no participants had received these vaccines, as only standard-dose, egg-based vaccines are included in the National Immunization Program (NIP). Considering the potential benefits of highly immunogenic vaccines, their inclusion in the NIP could be considered to improve overall influenza protection among at-risk populations.

Meta-analysis studies showed variations in the efficacy and effectiveness by vaccine types [19,20]. There are differences in both the types of influenza vaccines used and their distribution across countries. Compared to the interim VE reported by the CDC (36–51%) and Euro Surveillance (34–52%), the VE in South Korea was relatively lower [21,22]. In South Korea, influenza vaccination predominantly relies on the standard-dose egg-based vaccines, with coverage rates exceeding 80% each year. Some studies suggest that repeated administration of egg-based vaccines preferentially increases antibodies targeting egg-adapted epitopes, which may potentially reduce the immune response to circulating viruses [23,24]. In countries like South Korea, where more than 80% of the elderly receive standard-dose egg-based influenza vaccines annually, the potential impact of egg-adapted immune imprinting on reduced vaccine effectiveness may be relatively greater. Vaccine effectiveness (VE) in South Korea has tended to be lower than in other countries, not only in the current influenza season, but also in previous seasons with antigenic matching [25]. This suggests that country-specific factors might have a significant impact on VE even when antigenic matching occurs: vaccine types, high vaccine uptake rates, and high accessibility to the healthcare facilities in South Korea. First, in the United States, the United Kingdom, and Canada, high-dose or adjuvanted influenza vaccines were recommended and used for older adults, whereas in Korea, such high-immunogenicity influenza vaccines were not available during the study period, which may have influenced the assessment of vaccine effectiveness. Second, hybrid immunity is known to provide strong protective and preventive effects; however, high vaccination coverage may reduce opportunities for natural influenza exposure, which could lead to a relatively lower observed vaccine effectiveness [12]. In addition, high healthcare accessibility can lead to increased diagnosis of mild influenza cases, as more individuals with minor symptoms are likely to seek medical attention. This may result in an underestimation of influenza vaccine effectiveness, since milder cases are detected among vaccinated individuals.

This study had several limitations. First, the study population included a high proportion of older adults and individuals with underlying comorbidities, which may have contributed to the lower observed vaccine effectiveness [26,27]. Compared to the interim VE from our study last year, there were significant differences in the proportion of individuals aged 65 and older (25.2% vs. 34.7%) and those with underlying conditions (38.9% vs. 52.9%) [28]. Second, two different diagnostic methods (RAT and RT-qPCR) were used, which have different sensitivities and specificities. The lower sensitivity of RATs may have led to misclassification of influenza cases and subsequent underestimation of vaccine effectiveness, as reported in previous studies [29]. To address this potential bias and minimize false negatives, our study included only negative controls who underwent RATs within 48 h of symptom onset. In contrast to other countries where RT-qPCR is used exclusively for VE studies, RATs are commonly used in clinical settings in South Korea, particularly in outpatient care, due to practical considerations such as cost, accessibility, and turnaround time. While this may introduce measurement bias, the use of RATs reflects real-world practice and provides practical insights into vaccine performance in routine care. Given the limited sample size in the sensitivity analysis, meaningful conclusions could not be drawn, and the full-term analysis was therefore used for further evaluation. Third, most of the vaccines administered were standard-dose egg-based vaccines, and the proportion of high-immunogenicity vaccines (high-dose or adjuvanted vaccines) has been very low in South Korea. Therefore, it is difficult to evaluate vaccine effectiveness by the vaccine’s type. However, studies from other countries comparing immunogenicity and effectiveness across vaccine types suggest possible differences in vaccine effectiveness as well [17,18,23,30]. Finally, because of the small sample size, we were unable to perform subgroup analyses with sufficient statistical power to detect significant differences among influenza subtypes. Similarly, analyses stratified by comorbidities or age groups were also underpowered, limiting our ability to draw definitive conclusions in these subgroups. In high-risk groups, particularly older adults, non-significant findings may still indicate potential clinical relevance, as the vaccine could reduce the severity of illness. This limitation should be considered when interpreting the results, and larger studies are needed to confirm these findings. Additionally, as this study presents interim results from November to December 2024, the vaccine effectiveness estimates may not fully represent the entire influenza season. In the full-season analysis, we plan to conduct subgroup analyses stratified by diagnostic method (restricted to patients tested with RT-qPCR) and by vaccine type, to more accurately assess potential differences in vaccine effectiveness.

## 5. Conclusions

During the first two months of the 2024–2025 season, the effectiveness of the influenza vaccine was assessed as lower than expected but showed a statistically significant effect in preventing hospitalizations. A full-term analysis and genetic analysis of the virus are needed to identify the factors contributing to the lower effectiveness.

## Figures and Tables

**Table 1:** Baseline characteristics of the study participants.

	No. of Participants (n = 990)	Test-Positive Cases (n = 495)	Test-Negative Controls (n = 495)	*p*-Value †
**Month of enrollment**				1.000
November, 2023	20 (2.0)	10 (2.0)	10 (2.0)	
December, 2023	970 (98.0)	485 (98.0)	485 (98.0)	
**Age group**				1.000
19–64 years	646 (65.2)	323 (65.2)	323 (65.2)	
≥65 years	344 (34.7)	172 (34.7)	172 (34.7)	
**Sex**				0.749
Female	547 (55.3)	271 (54.7)	276 (55.8)	
Male	443 (44.7)	224 (45.3)	219 (44.2)	
**Diagnostic tests**				0.813
Rapid antigen test	880 (88.9)	426 (86.1)	454 (91.7)	
Polymerase chain reaction	239 (24.1)	102 (20.6)	137 (27.7)	
**Comorbidity**				
Diabetes mellitus	204 (20.6)	96 (19.4)	108 (21.8)	0.346
Cardiovascular disease	97 (9.8)	47 (9.5)	50 (10.1)	0.748
Chronic pulmonary disease	86 (8.7)	45 (9.1)	41 (8.3)	0.652
Chronic renal disease	74 (7.5)	39 (7.9)	35 (7.1)	0.629
Chronic liver disease	30 (3.0)	17 (3.4)	13 (2.6)	0.458
Chronic neurological disease	98 (9.9)	43 (8.7)	55 (11.1)	0.202
Solid malignancy	134 (13.5)	48 (9.7)	86 (17.4)	<0.001 *
Hematologic malignancy	35 (3.5)	15 (3.0)	20 (4.0)	0.390
Immunosuppressive agent use	31 (3.1)	13 (2.6)	18 (3.6)	0.362
Autoimmune disease	26 (2.6)	9 (1.8)	17 (3.4)	0.112
**Influenza vaccination, 2024/25 season**	401 (40.5)	205 (41.4)	196 (39.6)	0.560
**Clinical outcomes**				
Admission	382 (38.6)	152 (30.7)	230 (46.5)	<0.001 *
ICU Admission	88 (8.9)	39 (7.9)	49 (9.9)	0.588
Mechanical ventilation	41 (4.1)	18 (3.6)	23 (4.6)	0.684
Mortality	14 (1.4)	7 (1.4)	7 (1.4)	0.997

Abbreviations: VE, vaccine effectiveness. * *p* < 0.05. † *p* values were calculated by comparing test-positive and test-negative cases.

**Table 2:** Estimated influenza vaccine effectiveness by subtype and age group.

	Test-Positive, Vaccinated/Total (%)	Test-Negative, Vaccinated/Total (%)	Adjusted VE † (95% CI) (%)	*p*-Value
**Influenza**				
Overall	205/495 (41.4)	196/495 (39.6)	−0.5 (−34.0 to 24.6)	0.971
19–64 years	90/323 (27.9)	76/323 (23.5)	−15.3 (−66.7 to 20.3)	0.450
≥65 years	115/172 (66.9)	120/172 (69.8)	18.5 (−30.7 to 49.2)	0.397
**Influenza A**				
Overall	198/479 * (41.3)	196/495 (39.6)	0.4 (−33.2 to 25.5)	0.979
19–64 years	87/313 (27.8)	76/323 (23.5)	−14.5 (−66.2 to 21.1)	0.476
≥65 years	111/166 (66.9)	120/172 (69.8)	20.2 (−28.7 to 50.5)	0.355
**Influenza B**				
Overall	8/17 (47.1)	196/495 (39.6)	−34.4 (−318.8 to 56.9)	0.610
19–64 years	3/10 (30.0)	76/323 (23.5)	−49.7 (−554.2 to 65.8)	0.592
≥65 years	5/7 (71.4)	120/172 (69.8)	−71.9 (−1074.2 to 74.8)	0.581

Abbreviations: VE, vaccine effectiveness. * Number of influenza A positive cases (out of 495 total positive cases). † VE estimates were adjusted for comorbidities using conditional logistic regression.

**Table 3:** Estimated influenza vaccine effectiveness against severe outcomes.

	Case †, Vaccinated/Total (%)	Control †, Vaccinated/Total (%)	Adjusted VE ‡ (95% CI) (%)	*p*-Value
Admission	169/382 (44.2)	232/608 (38.2)	31.9 (3.5 to 51.9)	0.031 *
ICU Admission	37/88 (42.0)	341/847 (40.3)	37.8 (−4.5 to 63.0)	0.073
Mechanical ventilation	22/41 (53.7)	356/893 (39.9)	−17.8 (−141.9 to 42.6)	0.655
Mortality	7/14 (50.0)	387/965 (40.1)	17.3 (−178.2 to 75.4)	0.759

Abbreviations: ICU, intensive care unit; VE, vaccine effectiveness. * *p* < 0.05. † Totals may not sum to 990 due to missing data for some variables. ‡ VE estimates were adjusted for comorbidities using conditional logistic regression.

## Data Availability

The data presented in this study are available in this article and Supplementary Materials.

## Supplementary Materials

The following supporting information can be downloaded at: https://www.mdpi.com/article/10.3390/vaccines13111100/s1. Figure S1: Flowchart of participant selection and analysis; Figure S2: Bar chart on the overall distribution of influenza subtypes and RT-qPCR results. Distributions of influenza virus types determined by both rapid antigen testing and RT-qPCR (left), and subtypes determined by the RT-qPCR alone (right); Table S1: Estimated influenza vaccine effectiveness by age group among rapid antigen test (RT-qPCR) tested patients.

## Abbreviations

The following abbreviations are used in this manuscript:

VE	vaccine effectiveness
RSV	respiratory syncytial viruses
ILI	influenza-like illness
