# Supplementary material for: Interim Vaccine Effectiveness Against Influenza and Hospitalization, Republic of Korea, 2024–2025 (HIMM Network)
Source: Vaccines (Basel). 2025 Oct 28;13(11):1100. doi: 10.3390/vaccines13111100 (PMC12656372; doi:10.3390/vaccines13111100)
Supplement: Supplementary file 1 [file vaccines-13-01100-s001.zip › vaccines-3892397-supplementary.pdf]

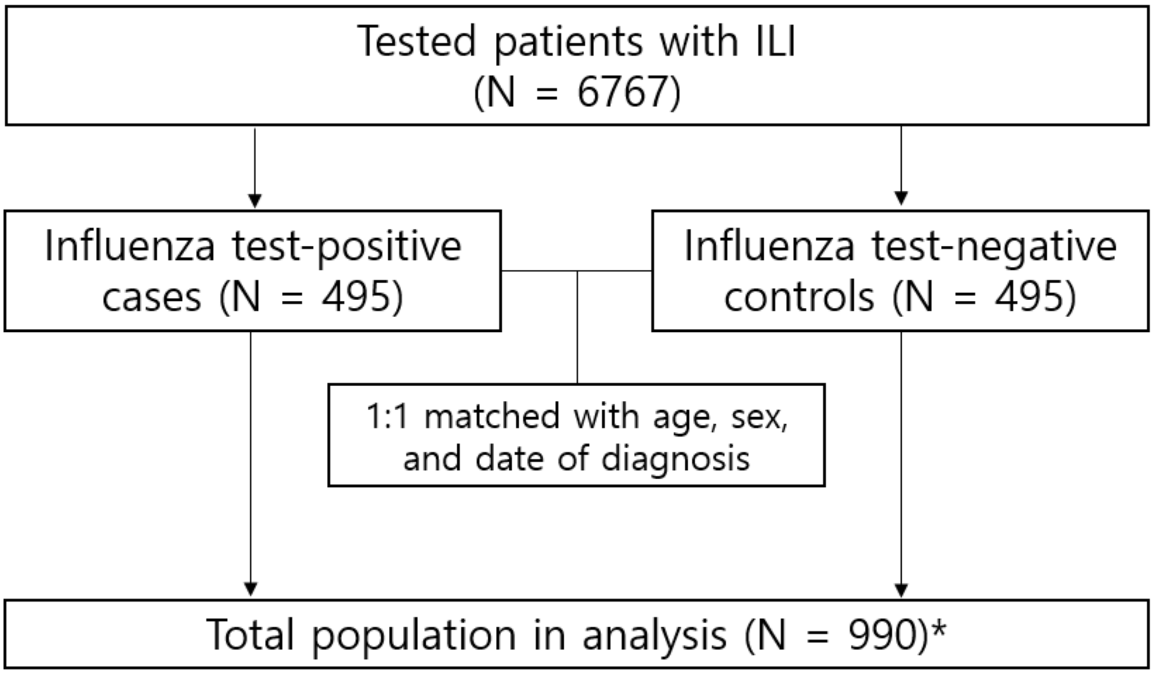

Supplementary Figure S1. Flowchart of participant selection and analysis.

* RAT was taken in 880 cases, and RT-qPCR was taken in 239 cases

ILI, influenza-like illness; RAT, rapid antigen test; RT-qPCR, reverse transcription quantitative polymerase chain reaction

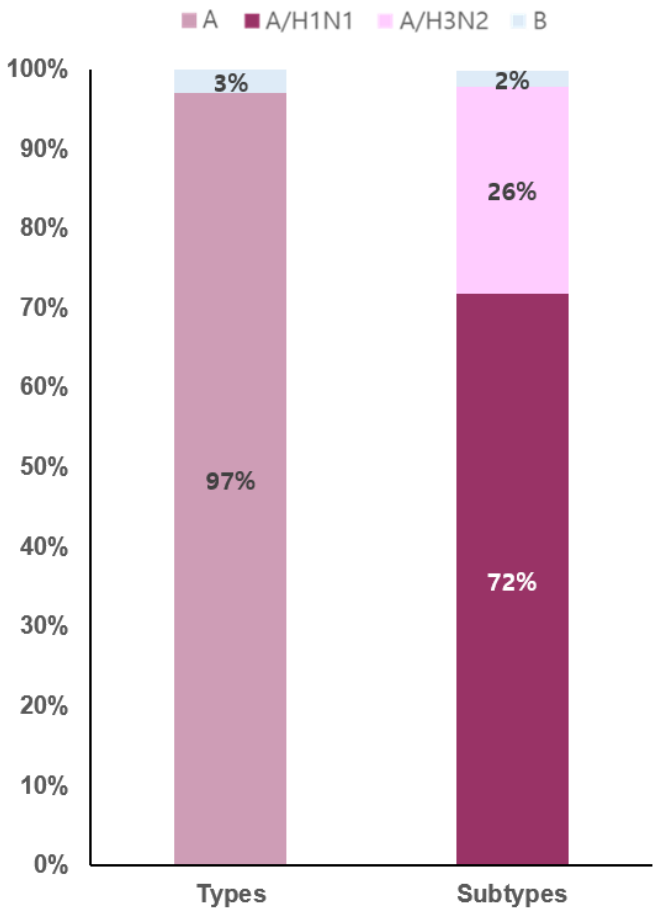

Supplementary Figure S2. Bar chart on the overall distribution of influenza subtypes and RT-qPCR results. Distributions of influenza virus types determined by both rapid antigen testing and RT-qPCR (left), and subtypes determined by the RT-qPCR alone (right).

Supplementary Table S1. Estimated influenza vaccine effectiveness by age group among rapid antigen test (RT-qPCR) tested patients.

	Test-positive, vaccinated/total (%)	Test-negative, vaccinated/total (%)	Adjusted VE (95% CI) (%)	*P*-value
**Influenza**				
Overall	47/102 (46.1)	45/137 (32.8)	-1.8 (-109.9 to 50.6)	0.961
19–64 yrs	3/39 (7.7)	10/84 (11.9)	50.6 (-115.1 to 88.7)	0.347
≥65 yrs	44/63 (69.8)	35/53 (66.0)	-28.1 (-212.6 to 47.5)	0.586
